# Comparative Transcriptomic Analyses of Vegetable and Grain Pea (*Pisum sativum* L.) Seed Development

**DOI:** 10.3389/fpls.2015.01039

**Published:** 2015-11-25

**Authors:** Na Liu, Guwen Zhang, Shengchun Xu, Weihua Mao, Qizan Hu, Yaming Gong

**Affiliations:** ^1^Institute of Vegetables, Zhejiang Academy of Agricultural SciencesHangzhou, China; ^2^Center of Analysis and Measurement, Zhejiang UniversityHangzhou, China

**Keywords:** transcriptome, high-throughput sequencing, grain pea, vegetable pea, seed development, sugar, starch

## Abstract

Understanding the molecular mechanisms regulating pea seed developmental process is extremely important for pea breeding. In this study, we used high-throughput RNA-Seq and bioinformatics analyses to examine the changes in gene expression during seed development in vegetable pea and grain pea, and compare the gene expression profiles of these two pea types. RNA-Seq generated 18.7 G of raw data, which were then *de novo* assembled into 77,273 unigenes with a mean length of 930 bp. Our results illustrate that transcriptional control during pea seed development is a highly coordinated process. There were 459 and 801 genes differentially expressed at early and late seed maturation stages between vegetable pea and grain pea, respectively. Soluble sugar and starch metabolism related genes were significantly activated during the development of pea seeds coinciding with the onset of accumulation of sugar and starch in the seeds. A comparative analysis of genes involved in sugar and starch biosynthesis in vegetable pea (high seed soluble sugar and low starch) and grain pea (high seed starch and low soluble sugar) revealed that differential expression of related genes at late development stages results in a negative correlation between soluble sugar and starch biosynthetic flux in vegetable and grain pea seeds. RNA-Seq data was validated by using real-time quantitative RT-PCR analysis for 30 randomly selected genes. To our knowledge, this work represents the first report of seed development transcriptomics in pea. The obtained results provide a foundation to support future efforts to unravel the underlying mechanisms that control the developmental biology of pea seeds, and serve as a valuable resource for improving pea breeding.

## Introduction

Pea (*Pisum sativum* L.) is one of the most widely grown grain legumes in the world, and its seeds are an important source of protein for human diets as well as for animal feed (Bastianelli et al., 1998). Pea seeds are rich in starch, soluble sugars, fiber, minerals, vitamins, and in secondary metabolites such as isoflavonoids with anti-cancer and other health-promoting activities (Dixon and Sumner, 2003). As such, improving the overall nutritional quality of pea seeds is an important and ongoing objective for pea breeding. Various studies on legume seed development show that the process is genetically programmed and correlated with changes in metabolite levels (Weber et al., 2005; Le et al., 2007). Despite this understanding, little molecular genetics work has been performed on pea, due to both the size of genome (4.5 Gb) as well as its highly repetitive nature (75–97%), causing it to be considered as a “genomic orphan” species (Macas et al., 2007; Smýkal et al., 2012). Such genomic information, however, is essential for deciphering the genetic control of pea seed development.

Seed development is a highly complex process and mainly divided into three stages—embryogenesis, expansion, and maturation—according to changes in seed weight (Baud et al., 2002; Gutierrez et al., 2007). The process includes transcriptional and physiological reprogramming reconciled by many different pathways, and involves the coordinated expression and regulation of thousands of genes (Gutierrez et al., 2007; Santos-Mendoza et al., 2008). A comprehensive earlier study on changes in gene expression during seed development in *Medicago truncatula* determined that a total of 15,786 genes were differentially expressed during seed maturation (Benedito et al., 2008). More than 45% of the functionally classified seed-regulated genes were assigned to metabolic pathways, including carbohydrate, amino acid, lipid, energy, and secondary metabolism, indicating that the seed development process is prone to metabolic control.

Over the past 10 years, several studies have integrated information from genetic programs and from both metabolic and hormone-responsive pathways, to provide evidence of the concerted action of a complex regulatory network triggering the seed development process (Weber et al., 2005; Gutierrez et al., 2007). Among metabolites, carbohydrates are known to represent a major carbon and energy source during seed development (Weber et al., 2005). Moreover, sucrose plays a dual role in the cell, as it is central in carbohydrate metabolism and acts as a signal molecule triggering storage-associated processes (Gibson, 2005). In addition to metabolic control, hormonal control is also important in seed development (Gibson, 2004). Previous studies by measurements of endogenous hormone concentrations in developing legume seeds have shown that cytokinin and gibberellins are primary requirement for the commencement of seed development, whereas auxin plays roles in establishing the embryonic body plan (McCarty, 1995) and abscisic acid (ABA) is critical for promoting embryo maturation (Finkelstein et al., 2002). Recent data has also highlighted a number of transcription factors that are specifically involved in the process of seed development, such as B3, MYB, bHLH, and AP2 (Sreenivasulu and Wobus, 2013). Together, these results illustrate the complexity of seed development regulation. Since grain legumes as crop plants are selected for high seed yield and characterized by high metabolic activity and fluxes in seeds, progress toward a better understanding of mechanisms controlling legume seed development is crucial. While these mechanisms have been widely studied in the model plants *M. truncatula* (Gallardo et al., 2007; Benedito et al., 2008) and soybean (Dhaubhadel et al., 2007; Severin et al., 2010; Jones and Vodkin, 2013), it is poorly understood in pea.

In order to explore the possibilities of improvement in the nutritional quality and quantity of pea seeds, it is necessary to have a clear understanding on the regulatory mechanisms involved in the process of seed development. Such an understanding may be best achieved through an exhaustive study of the transcriptome of the developing seed that may provide a blueprint gene expression networks underlying the seed development processes. In recent years, RNA sequencing (RNA-Seq) based on next-generation sequencing (NGS) allows studies to be conducted on species without a corresponding reference genome (Brautigam et al., 2011). RNA-Seq has been successfully adopted to study changes in gene expression during seed development in plants such as *Arabidopis* (Le et al., 2010), rice (Xue et al., 2012), and also in important legumes such as soybean (Severin et al., 2010; Jones and Vodkin, 2013), *M. truncatula* (Gallardo et al., 2007), and chickpea (Pradhan et al., 2014). In pea, there have been several studies reporting transcriptome analysis in recent years (Franssen et al., 2011; Kaur et al., 2012; Duarte et al., 2014; Sindhu et al., 2014; Sudheesh et al., 2015), mainly for use in the discovery of genetic markers. Recently, Alves-Carvalho and his colleagues announced a RNA-Seq data resource for pea including 20 cDNA libraries, which encompass various tissues harvested at different developmental stages and grown in contrasted N-nutritive conditions (Alves-Carvalho et al., 2015). However, none of the previous studies have focused specifically on pea seed development.

Food-oriented peas are sorted into either grain pea or vegetable pea, with large differences in morphology, nutrition, and taste between the two types. Vegetable peas are large-seeded pea cultivars that are harvested as immature seed. It typically has a higher content of sugar than grain pea, and is therefore sweeter. There are a large number of genes that affect carbohydrate synthesis in pea. Previous studies have reported that starch and sugar synthesis in pea is affected by mutant alleles at three loci, *r, rb*, and *bsg*, which are referred to starch branching enzyme (SBE), ADP-glucose pyrophosphorylase (AGPase), and phosphoglucomutase (PGM), respectively (Hedley et al., 1986; Hylton and Smith, 1992; Harrison et al., 2000). The seed accumulates a low level of starch and a high level of sucrose if it contains the *r* or *rb* genes or *bsg* gene within its genome (Lloyd et al., 1996; Harrison et al., 2000). The demand for vegetable peas as fresh or frozen seed is increasing in recent years as consumers perceive that sweeter peas have better flavor. Although seed development and nutrition accumulation are key biological processes that are of important economic and nutritional value in pea, the regulatory mechanisms remain elusive. To understand the genes and gene networks that play a role in regulating seed development and nutrition accumulation in grain and vegetable peas, we used RNA-Seq to generate and compare the transcriptomes of vegetable pea and grain pea at early and late developmental stages. Therefore, this study was particularly conducted (i) to determine changes in gene expression during seed development within pea types, and (ii) to compare gene expression at specific stages of seed development between two pea types. This study will provide a high-throughput sequencing and expression resource for future molecular studies on pea seed development.

## Materials and methods

### Plant material

**Figure 1:**
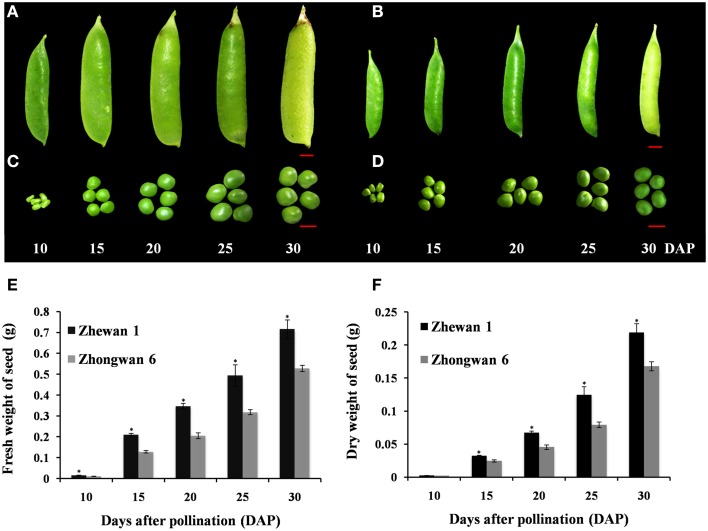
**Images of pea pods and seed developmental stages, and seed weight measurements**. The pod and seed of the vegetable pea Zhewan 1 **(A,C)** and the grain pea Zhongwan 6 **(B,D)** at 10, 15, 20, 25, and 30 days after pollination (DAP). Seeds at 10 and 25 DAP of the two cultivars were submitted for transcriptome sequencing. The fresh and dry weights of pea seeds at each point in time are shown in **(E,F)**. Mean weight in **(E,F)** are shown with standard errors bars from three repeated experiments. Asterisk indicates that weight is significantly different between Zhewan 1 and Zhongwan 6 according to a LSD test at *P* < 0.05. Red bar = 1 cm.

The vegetable pea cultivar “Zhewan 1” and the grain pea cultivar “Zhongwan 6” were selected as materials in the current study. These two cultivars are widely used for commercial production in China and they represent typical features of vegetable pea and grain pea. In this study, they were grown in the field of the Vegetable Research Institute, Zhejiang Academy of Agricultural Sciences. Peas are self-pollination species, and the first day the flower becomes fully open is usually considered the first day of fertilization (Gepts et al., 2005). The flowers of these two cultivars were tagged on the day they opened completely. In Figure 1, the different developmental stages of the seeds of these two pea cultivars are shown. The uniform pea pods samples were collected at different developmental stages (10, 15, 20, 25, and 30 days after pollination [DAP]). Ten DAP corresponds to an early stage (cell division and morphogenesis); 15, 20, and 25 DAP correspond to middle stages (cell expansion and seed filling); and 30 DAP corresponds to physiological maturity. To control the effect of biological variation, the materials that were collected for each sample were composed of large amounts from many individuals. The seeds from different plants were pooled together as one biological sample and three biological replicates were collected for each sample. The samples were frozen in liquid nitrogen immediately and stored at −80°C until further use.

### RNA extraction, library construction, and RNA-seq

Total RNA was extracted with TRIzol Reagent (Invitrogen, USA) according to the manufacturer's instructions, and DNase I was used to clean out DNA. Separate RNA samples from two different developmental stages, 10 and 25 DAP, of the seeds of Zhewan 1 and Zhongwan 6 were constructed for RNA-Seq. These two stages represent the early developmental stage and the harvest stage of the vegetable pea. The experiment was performed with three biological replicates for each of the two seed developmental stages and pea types. The four libraries are indicated as “Zhewan 1–10,” “Zhewan 1–25,” “Zhongwan 6–10,” and “Zhongwan 6–25.” The following protocols were performed by LC Science (Hangzhou, China). The quality and integrity of the RNA was examined using an Agilent 2100 Bioanalyzer (Agilent Technologies, USA). The mRNA was isolated using oligo (dT)-attached magnetic beads and was broken into short fragments (approximately 200 bp). Using these short fragments as templates, the first-strand cDNA and the second-strand cDNA were synthesized. Sequencing adapters were ligated to the short fragments after purifying with the QiaQuick PCR extraction kit (Qiagen, The Netherlands), which were used to distinguish different sequencing samples. The required fragments were then separated by agarose gel electrophoresis and selected for PCR amplification as sequencing templates. Finally, the libraries were sequenced using an Illumina HiSeq™ 2000 (San Diego, CA, USA). The sequence data has been deposited into the NCBI/GEO database with accession number GSE72573.

### De novo assembly

Raw reads were first filtered by removing adapter sequences and low quality sequences, which included reads with N percentages (i.e., the percentage of nucleotides in a read that could not be sequenced) over 5% and ones that contained more than 20% of the nucleotides in the reads with a *Q* ≤ 10. The *Q*-value represented the sequencing quality of the related nucleotides. The clean reads were then *de novo* assembled using the Trinity assembly program (Grabherr et al., 2011).

### Gene annotation

The functions of the unigenes were annotated by BLASTing with an *E*-value threshold of 10^−5^ to protein databases that included the NCBI non-redundant (nr) database, the Swiss-Prot protein database, the Kyoto Encyclopedia of Genes and Genomes (KEGG) database, the Clusters of Orthologous Groups of proteins (COG) database, and the pea RNA-Seq gene atlas (http://bios.dijon.inra.fr/FATAL/cgi/pscam.cgi). Proteins with the highest sequence similarity were retrieved for the analyses. KEGG was used to produce an annotation of the metabolic pathways, while COG was used to match each annotated sequence to an ancient conserved domain, which represents the primary phylogenetic lineages of the pea. Based on the nr annotation, 6 top-hit species were identified, and Gene Ontology (GO) classifications were obtained with Blast2GO. Transcript diversity and abundance were scrutinized with MapMan (Thimm et al., 2004) using the *M. truncatula* peptide database downloaded from MapMan as reference. Accordingly, pea seed transcripts were first BLASTed (*E* ≤ 1E-05) with the *M. truncatula* peptide database downloaded from the ftp site of Phytozome v9.1.

### Differentially expressed gene analysis

The alignment software, bowtie 0.12.8, was used to map the reads back to the transcriptome. The number of mapped clean reads for each unigene was then counted and normalized into a RPKM value (Reads Per kb per Million reads), which is widely used to calculate the unigene expression (Mortazavi et al., 2008). To detect differentially expressed genes, statistical analyses among libraries were performed following the formula as described (Shen et al., 2012), where the false discovery rate (FDR) used to determine the *P*-value threshold in multiple tests and analyses was calculated using the *q*-value package (Benjamini et al., 2001). For this study, differentially expressed unigenes between two samples were screened with a threshold of FDR ≤ 0.001 and an absolute value of log2Ratio ≥ 1 (Feng et al., 2012; Sun et al., 2015; Wang et al., 2015). Furthermore, GO classifications and the KEGG pathway enrichments were compared between up-regulated and down-regulated unigenes.

### Real-time quantitative RT-PCR

Total RNA used for quantitative RT-PCR (qRT-PCR) analysis was extracted from the different developmental stages of the seeds of the two pea cultivars with three biological replicates using the same method as described above. For qRT-PCR, approximately 3 μg of total RNA was digested using RNase-free DNase I (TaKaRa, Kyoto, Japan) at 37°C to remove any genomic DNA. The digested RNA was then reverse-transcribed to cDNA using MMLV transcriptase (TaKaRa, Kyoto, Japan) in a 20 μl reaction. The cDNAs were then used as templates in qRT-PCR reactions using the SYBR Green Master Mix (TaKaRa, Kyoto, Japan) with gene-specific primers. The reactions were performed on an ABI StepOnePlus™ cycler (Applied Biosystems, USA). The two-step quantitative RT-PCR program began at 95°C for 30 s, followed by 40 cycles of 95°C for 5 s and 60°C for 20 s. Fluorescent signals were collected at each polymerization step. Expression was calculated as 2^−ΔΔCt^ (Livak and Schmittgen, 2001) and was normalized to that of the control gene β-tubulin (Die et al., 2010). The primers used for qRT-PCR are listed in Supplementary Table S1. The correlation between log2 normalized qRT-PCR and RPKM values was plotted using Origin 8 (OriginLab, USA).

### Fresh weight and dry weight assessment

For each time point, fresh and dry weights of the seeds were determined after sample collection. The seeds were removed from the pods immediately after sampling, and fresh weights were recorded. The seeds were then dried in a forced-air oven at 70°C until the weight remained constant, and dry weights were recorded. Each sample contained 100 seeds and was determined in triplicate.

### Measurements of sugar and starch

The extraction of sugars was performed according to previous methodology with minor modifications (Zhang et al., 2005). Briefly, 2 g of the sample stored at −80°C was ground into fine powder in liquid nitrogen with a Grinder CK 1000 (Thmorgan, China), and then homogenized in 6.0 ml of an ethanol (80%) solution, shaken for 40 min at 80°C, and centrifuged at 10,000 rpm for 10 min. The supernatant was collected, and the precipitate was homogenized again with ethanol (80%). This procedure was repeated; the supernatants were prepared to a constant volume of 25 ml. Three milliliter of extract was dried into powder with a Termovap Sample Concentrator (Eyela, Japan), and the volume was prepared to 1 ml with distilled water and used for sugar analysis. Soluble sugars were analyzed with HPLC (Waters, USA), following the method of Koch and Avigne (1990). Water was used as the mobile phase at a flow rate of 0.5 ml/min. A NH_2_ (4.6 mm × 250 mm) column (GL Scoemces Inc., Japan) and a refractive index detector RI-1530 (Jasco, Japan) were used. The concentrations of starch were measured with traditional anthrone colorimetry. Fully mixed frozen seeds (0.2 g) were prepared for each sample. The amylose contents were evaluated with a dual-wavelength iodine binding colorimetry method as previously described (Nakkanong et al., 2012).

## Results and discussion

### RNA-seq and de novo assembly of grain pea and vegetable pea

Pea pods from the vegetable pea Zhewan 1 and the grain pea Zhongwan 6 were sampled at 10–30 days after pollination with 5-day intervals during seed development. From measurements across five stages, the fresh and dry seed weights of Zhongwan 6 throughout seed development were approximately 70% that of Zhewan 1 (Figure 1). Because we are interested in understanding the transcriptional changes that may be involved in regulating pea seed development and the differences between grain pea and vegetable pea, we performed RNA-Seq analyses of early and late developing seeds of Zhewan 1 and Zhongwan 6. Four libraries were generated from RNA that was isolated from seeds of the two cultivars at 10 and 25 days after pollination, which was then paired-end sequenced using the Illumina platform.

Each library produced numbers of raw reads that ranged from 38.7 to 53.5 M (Table 1). After cleaning and quality checks, approximately 38.6–53.5 M high quality reads were obtained, with a validity ratio of 99.96%. In total, 186,660,706 reads of 100 bp were generated, giving a total of over 18 billion nucleotides of sequence data. The data produced from the four libraries were pooled together for more comprehensive reconstruction of the transcriptome, which were then used as the reference to facilitate comparison of individual samples for further gene expression analysis.

**Table 1:** **Summary of the RNA-Seq data collected from the vegetable pea Zhewan 1 and the grain pea Zhongwan 6 at 10 and 25 DAP seed developmental stages**.

**Category**	**Vegetable pea**		**Grain pea**	
	**10 DAP**	**25 DAP**	**10 DAP**	**25 DAP**
Raw sequencing reads	38,650,844	45,082,448	49,483,656	53,517,106
Clean reads	38,635,654	45,064,114	49,464,658	53,496,280
Raw bases	3,865,084,400	4,508,244,800	4,948,365,600	5,351,710,600
Clean bases	3,863,565,400	4,506,411,400	4,946,465,800	5,349,628,000

**Figure 2:**
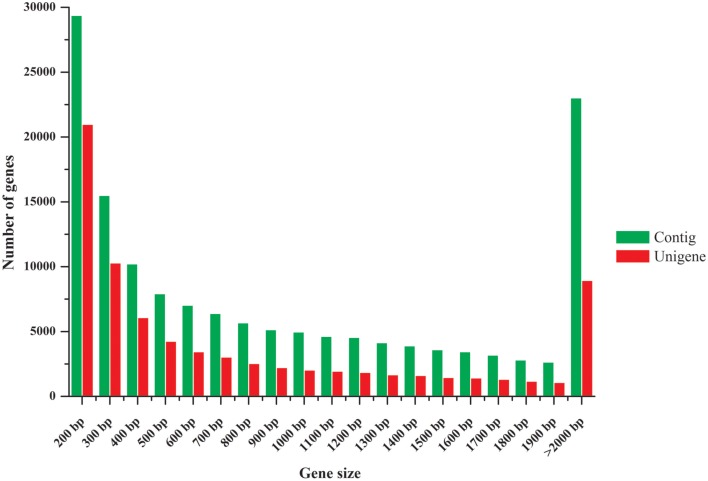
**Length distribution of assembled contigs and unigenes**.

The clean raw reads were assembled into 148,081 contigs with a mean length of 1117 bp. Using paired-end joining and gap-filling, these contigs were further assembled into 77,273 unigenes with a mean length of 930 bp. A N50 length of 1603 bp, i.e., half of the assembled bases were incorporated into unigenes with a length at least 1603 bp, was obtained. The size distributions of these contigs and unigenes are shown in Figure 2. Previous pea transcriptome assemblies using Roche 454 technology reported an average contig length of 454 bp (Franssen et al., 2011), 719 bp (Kaur et al., 2012), and 842 bp (Duarte et al., 2014). Here, we obtained a much longer average contig length (1117 bp) than those in previous studies. Furthermore, a comparison of our assembly to the previous studies showed that it covered them well, with 13,322 out of 13,445 contigs (99%) from Kaur et al. (2012), 79,231 out of 81,449 (97%) from Franssen et al. (2011), 66,618 out of 68,850 (97%) from Duarte et al. (2014), and 41,141 out of 46,099 (89%) from Alves-Carvalho et al. (2015) having a hit against our assembly (megablast with e-value lower than 1e-5). Reciprocally, from 77,273 unigenes from our study, 34,663 (45%), 26,256 (34%), 48,637 (63%), and 58,850 (76%) had a hit against Franssen et al. (2011), Kaur et al. (2012), Duarte et al. (2014) and Alves-Carvalho et al. (2015) assemblies, respectively. The much higher sequence coverage in our study enabled a greater degree of accuracy in sequence assembly and expression profiling analysis.

### Gene annotation

**Figure 3:**
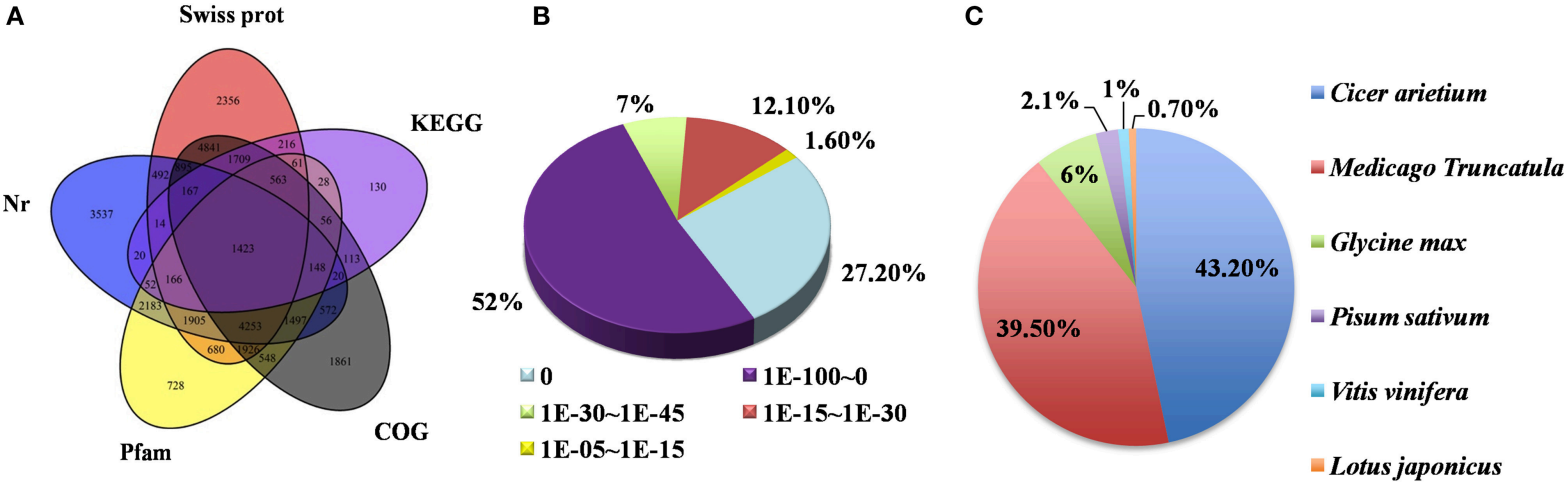
**Characteristics of homology search of pea unigenes**. **(A)** Venn diagram of the number of unigenes annotated by BLASTx with an *E*-value threshold of 10^−5^ against protein databases. The number in the circles indicate the number of unigenes annotated by single or multiple databases. **(B)** *E*-value distribution of the top BLASTx hits against the nr database for each unigene. **(C)** Percentage of unigenes matching the top 6 species using BLASTx in the nr database.

Unigenes were annotated based on BLASTx (cutoff E-value 10^−5^) searches of five public databases (Figure 3A). Among these databases, 38,887 unigenes were annotated to the nr database, 21,667 unigenes to the Swiss-prot database, 29,055 unigenes to the Pfam database, and 33,888 and 30,119 unigenes to the KEGG and COG databases, respectively (Figure 3A). Based on the nr annotation and the *E*-value distribution, 86.2% of the mapped sequences showed strong homology (*E* < 10^−30^) and 79.2% showed very strong homology (*E* < 10^−100^) to the available plant sequences (Figure 3B). With respect to species, 43.2 and 39.5% of the unique sequences had top matches to sequences from *Cicer arietinum* and *Medicago truncatula*, respectively, with additional hits to *Glycine max* (6%), *Pisum sativum* (2.1%), *Vitis vinifera* (1%), and *Lotus japonicas* (0.7%) (Figure 3C). This result is consistent with recent report on pea transcriptome analysis (Sudheesh et al., 2015). Taking advantage of the genome sequence of the closely related model species *M. truncatula* and the recently announced public pea (cv. “Cameor”) Unigene set online (http://bios.dijon.inra.fr/FATAL/cgi/pscam.cgi), our assembled unigenes were further aligned with these two data sets (Supplementary Tables S2, S3). The annotated unigenes provide an excellent platform for future genetic and functional genomic research in pea.

We used GO assignments to classify the functions of the predicted unigenes. GO annotated unigenes were categorized into three ontologies: biological process, cellular component, and molecular function. The molecular function category had the greatest number of unigenes included (32,399), followed by biological process (31,826) and then cellular component (21,960). Within the molecular function category, “ATP binding,” “DNA binding,” and “protein binding” were the most enriched, while proteins related to “regulation of transcription, DNA-dependent” and “transport” were enriched in the biological process category. Among the cellular component category, “integral to membrane” and “nucleus” accounted for most of the unigenes (Supplementary Table S4).

Furthermore, 23,059 unigenes with annotations were assigned to COG classifications. Among the 25 COG categories, the cluster for “general function prediction only” represented the largest category (4677, 20.3%), followed by “post-translational modification, protein turnover, chaperones” (2156, 9.3%), “signal transduction mechanisms” (2085, 9.0%), and “carbohydrate transport and metabolism” (1244, 5.4%), which confirmed that the development of pea seed involved complex metabolic and regulatory processes (Supplementary Figure S1). Furthermore, 8387 unigenes with annotation could be distributed into 229 KEGG pathways. Genes related to “ribosome” were found to be most abundant in number (310), followed by those for “oxidative phosphorylation” (176) and “glycolysis/gluconeogenesis” (137) (Supplementary Table S5).

### Comparison of gene expression of developing seeds 10 and 25 DAP for vegetable pea and grain pea

**Figure 4:**
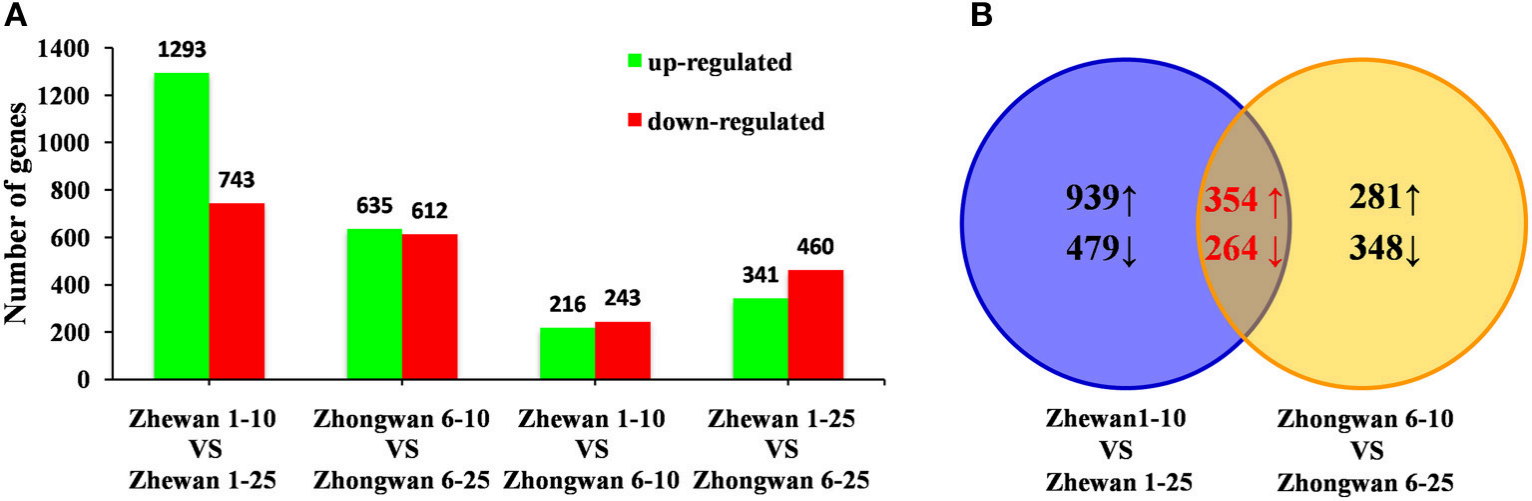
**Changes in gene expression profiles during seed development of the two pea cultivars**. **(A)** The number of up- and down-regulated genes in comparisons of the Zhewan 1–10 vs. Zhewan 1–25; Zhongwan 6–10 vs. Zhongwan 6–25; Zhewan 1–10 vs. Zhongwan 6–10; and Zhewan 1–25 vs. Zhongwan 6–25 are summarized. **(B)** VENN diagrams of DEGs from Zhewan 1–10 vs. Zhewan 1–25 and Zhongwan 6–10 vs. Zhongwan 6–25. The numbers marked in the diagram are the number of common genes significantly up- and down-regulated between the two sets (log2-fold change ≥ 1 and FDR ≤ 0.001).

We required a two-fold or greater change and FDR of 0.001 or less to identify differentially expressed genes (Figure 4A; Supplementary Tables S6–S9). Based on this stringency, we identified 2036 unigenes that were significantly differentially expressed in 25 DAP seeds compared with 10 DAP seeds in vegetable pea Zhewan 1 (Figure 4A); of these, 1293 were up-regulated and 743 were down-regulated. This is a greater number than in Zhongwan 6, where a total of 1247 unigenes were differentially expressed between the 10 and 25 DAP seeds, with 635 up-regulated and 612 down-regulated (Figure 4A). However, among these, 354 and 264 unigenes were up- and down-regulated respectively in both pairwise comparisons (Figure 4B; Supplementary Table S10). This suggests that some genes respond in the same way during seed development in both vegetable pea and grain pea. Comparing between the two cultivars, we discovered that there were many more differentially expressed genes in the late developmental stage than in the early stage. As shown in Figure 4A, between Zhongwan 6 and Zhewan 1 there were 801 (341 up-regulated and 460 down-regulated) unigenes that were significantly differentially expressed at 25 DAP, while only 459 (216 up-regulated and 243 down-regulated) unigenes were significantly differentially expressed at 10 DAP. The top 500 highly expressed genes differing between early and late seed developmental stages in Zhewan 1 and Zhongwan 6 were shown in Supplementary Figure S2.

### Validation of RNA-seq data by qRT-PCR

**Figure 5:**
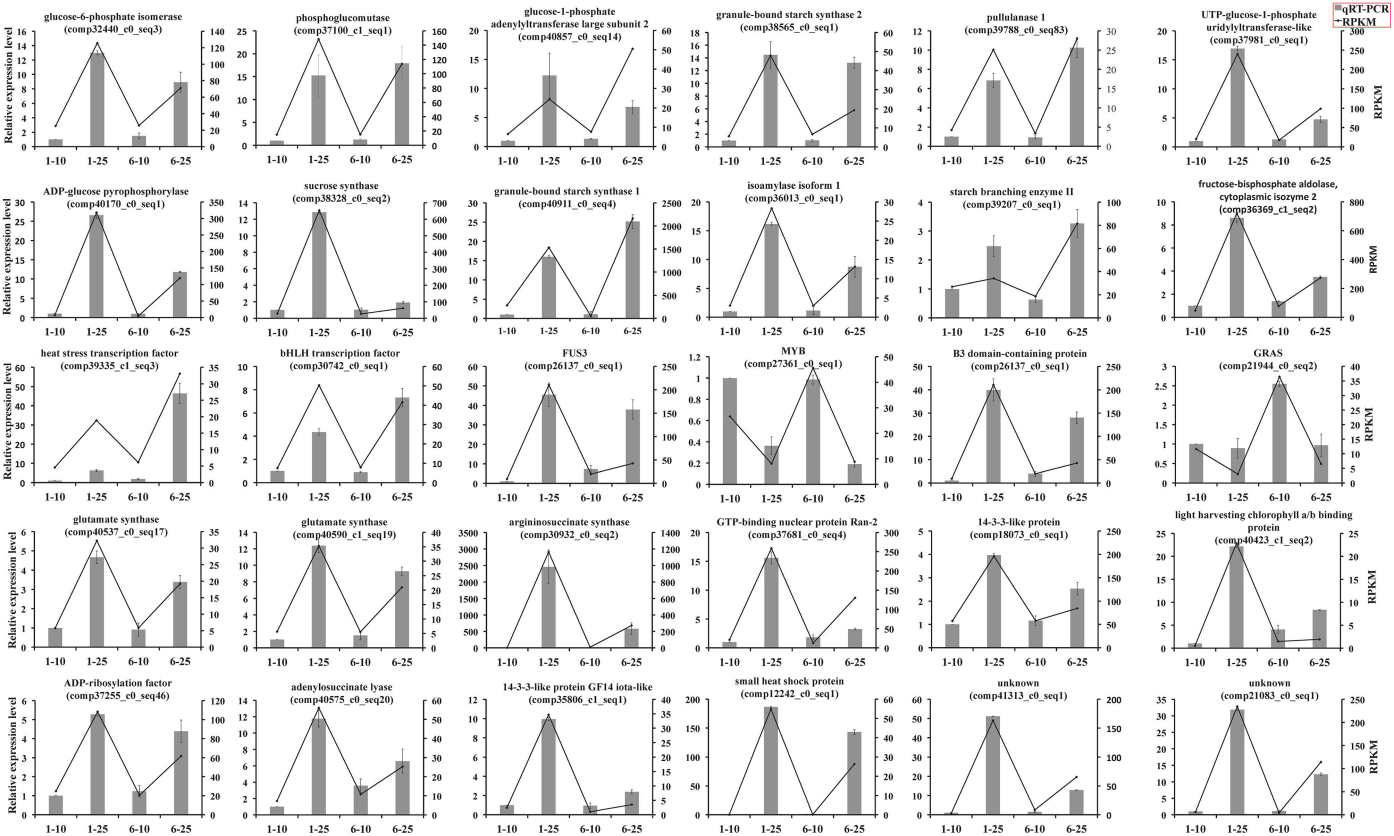
**qRT-PCR analysis of 30 differential expressed unigenes during seed development of vegetable pea and grain pea**. The expression patterns of selected genes were analyzed at 10 and 25 DAP. Black bars with standard errors represent the relative expression level determined by qRT-PCR from three independent biological replicateds (left y-axis). Lines indicate transcript abundance change based on RPKM values according to RNA-Seq (right y-axis).

To verify the differentially expressed genes identified by RNA-Seq, we performed qRT-PCR assays with independently collected samples from 10 to 25 DAP seeds from Zhewan 1 and Zhongwan 6 that were in the same developmental stages as those used for RNA-Seq. We randomly selected 30 unigenes with various degrees of expression to validate the RNA-Seq data. qRT-PCR data for these genes were basically consistent with RNA-Seq results (Figure 5). From linear regression analysis [(RT-PCR value) = a (RNA-Seq value) + b] the correlation coefficient was 0.8149, indicating a positive correlation between RNA-Seq data and qRT-PCR data (Supplementary Figure S3). Although the results showed that the exact fold changes varied between qRT-PCR and RNA-Seq, possibly due to differences in the sensitivity and specificity between the two approaches, the gene expression trends are similar. This indicates that the RNA-Seq data here are valuable, as seen in other studies (Pradhan et al., 2014; Guo et al., 2015; Lai et al., 2015; Zhao et al., 2015).

### Functional analysis of differently expressed genes

We used GO and KEGG assignments to classify the functions of the differentially expressed genes found in pairwise comparisons between the two stages of seed development in the grain and vegetable pea (Supplementary Figures S4, S5). In the biological process category, “translation” and “regulation of transcription, DNA-dependent” were significantly enriched in all four pairwise comparisons, whereas “photosynthesis,” “carbohydrate metabolic process,” and “glycolysis” were enriched in all comparisons except between Zhewan 1–10 and Zhongwan 6–10 (Supplementary Figure S4). For the KEGG pathways, the specific enrichment of unigenes was observed for several pathways that were involved in metabolic processes, such as glycolysis/gluconeogenesis, starch and sucrose metabolism, amino acid metabolism, and hormone metabolism (Supplementary Figure S5).

For a visual comparison of unigenes differentially expressed between early and late seed developmental stages in Zhewan 1 and Zhongwan 6, the data was then subjected to MapMan (Thimm et al., 2004; Sreenivasulu et al., 2008) (Supplementary Table S11). This allowed exploration of the global activation of specific metabolic pathways and gene regulatory networks activated during pea seed development. As shown in Supplementary Figures S6, S7, expression of unigenes related to carbohydrate metabolism (major and minor CHO metabolism), amino acid turnover, photosynthesis, tricarboxylic acid cycle (TCA), glycolysis, fermentation, and secondary metabolism are generally increased at 25 DAP compared to at 10 DAP seeds in both cultivars. For example, up-regulation of genes involved in amino acid metabolism during seed development was observed in both vegetable pea and grain pea (Supplementary Figure S8A; Supplementary Table S12). Increased expression pattern of these genes is consistent with intensive amino acid synthesis at seed filling stage (Gallardo et al., 2007; Benedito et al., 2008), which is essential for protein accumulation in pea seeds. Additionally, “photosynthesis” pathway was significantly enriched. We found all of the 70 and 40 unigenes associated with photosynthesis were significantly up-regulated at 25 DAP compared with 10 DAP in Zhewan 1 and Zhongwan 6 seeds, respectively (Supplementary Figure S8B; Supplementary Table S13). The main role of seed photosynthesis is reported to increase internal O_2_ content and to control biosynthetic fluxes by improving energy supply (Borisjuk et al., 2004), it can also affect the metabolism in a number of distinct ways (Ruuska et al., 2004). Our results indicate that many metabolic genes are most active during pea seed filling, which aligns with previous studies on *M. truncatula* where approximately half of the seed-regulated genes were assigned to metabolic pathways (Benedito et al., 2008).

Furthermore, a complex regulatory network triggers initiation of seed development, maturation and accumulation of storage products (Supplementary Figure S6). Hormones play crucial roles in seed development (Holdsworth et al., 2008), and detailed analysis revealed both up-regulation and down-regulation of hormone metabolism and response related genes (Supplementary Table S14). These results suggest that hormone levels should be dynamic, due to changing expression of hormone synthesis genes. Among them, most of the genes involved in auxin biosynthesis and signal transduction were down-regulated at late stage when compared to early stage (Supplementary Figure S8C), which suggests that auxin should mainly function at the early seed development stage. This is supported by a previous study in field pea, which showed that auxin concentration in developing pea seeds peaked at 8–12 DAP, dropping afterward (Slater et al., 2013). Overall, these results indicate that seed development is genetically programmed, correlated to increased metabolic activities, and governed by complex gene regulatory networks.

### Comparison of the sugar metabolism related gene expression at early and late developmental stages of Zhewan 1 and Zhongwan 6 seeds

**Figure 6:**
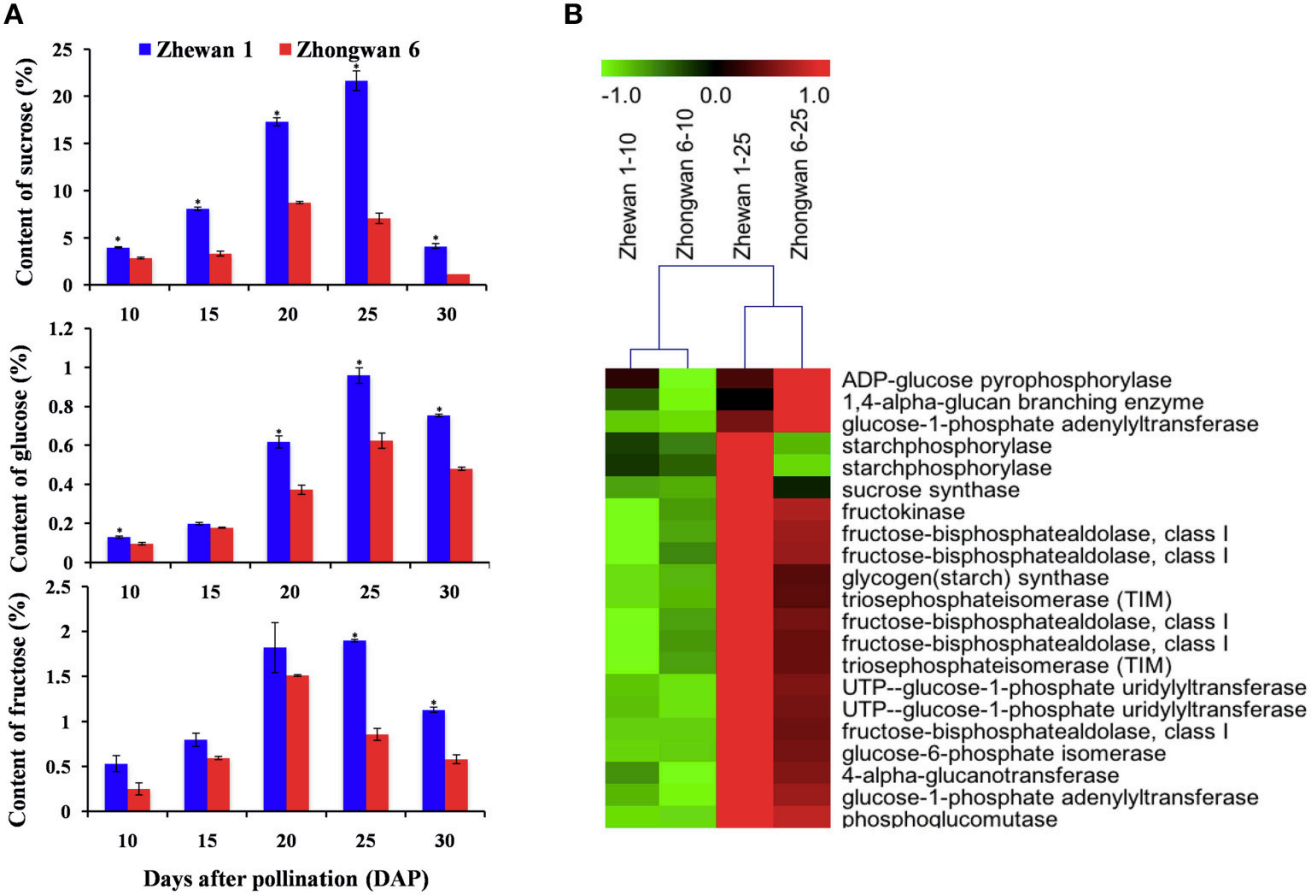
**Contents of sugars (sucrose, glucose and fructose) (A) and differential expression of genes for sugar biosynthesis (B) in Zhewan 1 and Zhongwan 6 during seed development**. Mean sugar contents are shown with standard errors bars from three repeated experiments. Asterisk indicates that sugar content is significantly different between Zhewan 1 and Zhongwan 6 according to an LSD test at *P* < 0.05.

The most noticeable difference in taste between vegetable pea and grain pea is the increased sweetness of vegetable pea over grain pea, with sugar providing the most important contribution. We measured soluble sugar content using HPLC in Zhewan 1 and Zhongwan 6 during seed development. As shown in Figure 6A, sucrose, which accounts for approximately 80% of the total soluble sugars in pea, increased from 10 to 25 DAP, peaking at 20 to 25 DAP, and decreased at 30 DAP in both cultivars (Figure 6A). Consistent with this, a previous study of *Vicia faba* seed showed that the sucrose content increased from 10 to 15 DAP, reached a peak around 25 DAP, and decreased at later developmental stages (Heim et al., 1993). However, there is a significant difference in sugar accumulation between pea and *M. truncatula* seeds, where the sucrose concentration peaked around 13 DAP and then gradually decreased up until maturity (Djemel et al., 2005). The diversity of seed sugar accumulation patterns between different legume species implies that distinct mechanisms should be involved. Importantly, we also found that sugar content in the seeds of Zhewan 1 was much higher than in Zhongwan 6 throughout the seed development process (for example, 21.7 vs. 7.1% at 25 DAP, respectively) (Figure 6A), which would explain why Zhewan 1 is much sweeter than Zhongwan 6.

A comparison of genes involved in sugar biosynthesis at early and late seed developmental stages in both cultivars was then conducted. More than 20 unigenes involved in sugar metabolism showed a dramatic increase in expression at 25 DAP seeds compared to 10 DAP seeds in both of Zhewan 1 and Zhongwan 6 (Figure 6B; Supplementary Table S15), in parallel with the physiological data and coinciding with the onset of accumulation of sugar in the developing pea seeds. This high expression of genes involved in sugar metabolism during seed development is consistent with transcriptomic analyses performed in rice and chickpea (Xu et al., 2012; Pradhan et al., 2014), which found that unigenes related to carbohydrate metabolism were more highly expressed at later stages compared to early stages. Previous work in legume and *Arabidopsis* seeds suggested that sugars mediate the switch from cell division to maturation (Wobus and Weber, 1999; Gibson, 2004). Our results provide evidence that the sugar metabolism process occurring in the pea seed should also involve a transition from cell growth and differentiation to seed development and maturation.

Because the content of soluble sugar is much higher in Zhewan 1 than that in Zhongwan 6 (Figure 6A), we further compared the expression level of genes participating in sugar metabolism between these two cultivars. The expression of these genes was not significantly different at 10 DAP. However, most of the sugar metabolism related genes such as TIM (triosephosphateisomerase), UgpA (uridylyltransferase), and fructose-bisphosphatealdolase had much lower expression levels in Zhongwan 6 than in Zhewan 1 seeds at 25 DAP (approximately one-fourth the level). The expression level of phosphoglucomutase (PGM), which is responsible for the conversion of glucose-6-phosphate to glucose-1-phosphate that affects the content of sucrose in pea (Harrison et al., 2000), was not significantly different between these two cultivars. Our results indicate that the sugar metabolism process is coordinated by several genes, and that differences in the level of seed sugar content between vegetable pea and grain pea could be the result of differential expression of these genes during the filling stage of seed development.

### Transcriptional regulation of starch biosynthesis in Zhewan 1 and Zhongwan 6 developing seeds

**Figure 7:**
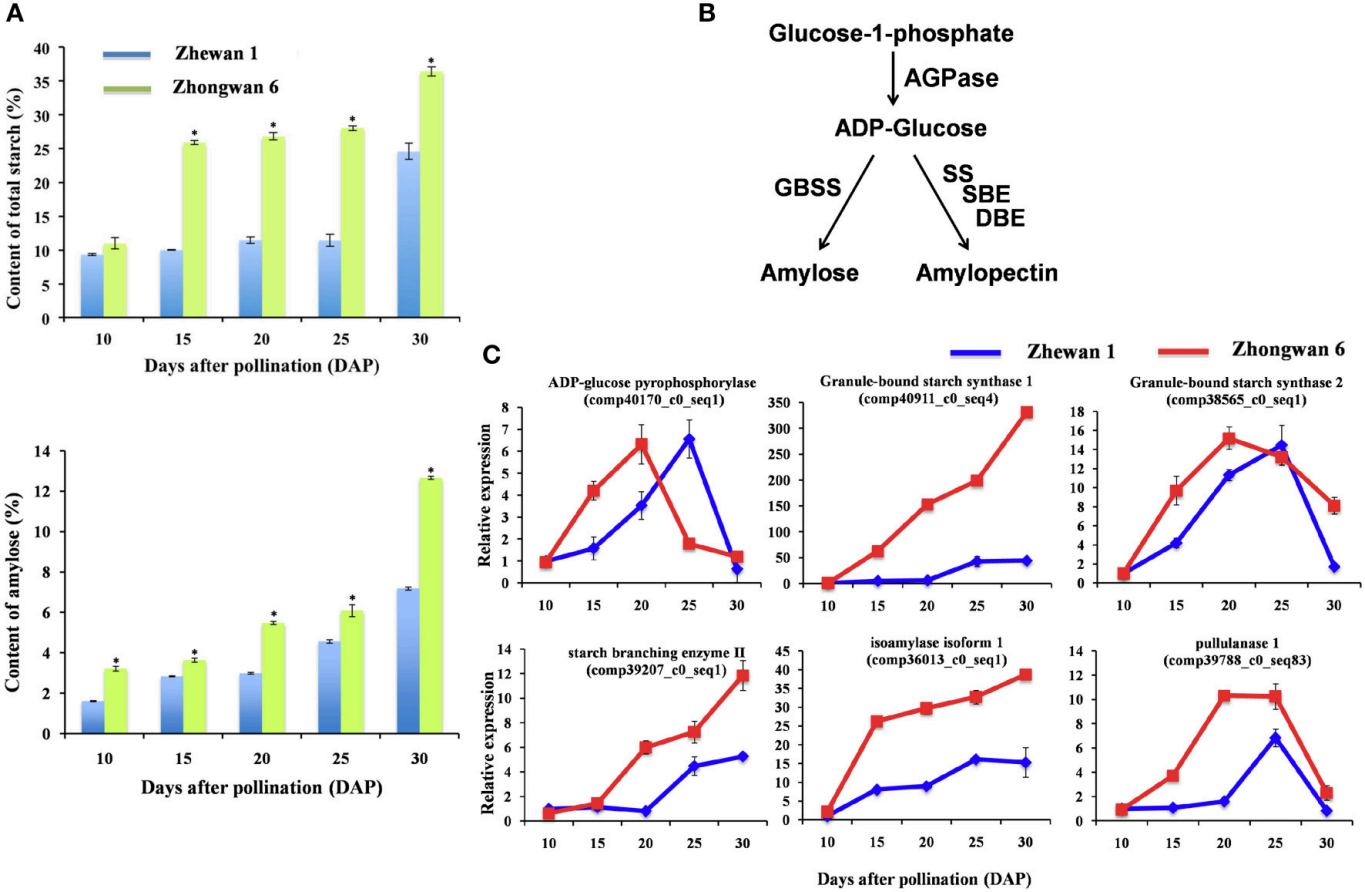
**Total content of starch and amylose in Zhewan 1 and Zhongwan 6 seeds during development (A), schematic of starch synthesis pathway (B) and qRT-PCR analysis of candidate differentially expressed genes related to starch biosynthesis (C)**. Mean starch and amylose contents in **(A)** is shown with standard errors bars from three repeated experiments. Asterisk indicates that starch and amylose contents are significantly different between Zhewan 1 and Zhongwan 6 according to an LSD test at *P* < 0.05.

During development, pea seeds also undergo a rapid change in starch content, which is directly related to the quality and taste of the pea. The molecular structure of plant starch consists almost completely of the glucose homopolymers amylopectin and amylase (Tetlow, 2006; Jeon et al., 2010). The amounts and proportions of amylose and amylopectin in plants vary depending on the type of storage organ, plant species, and cultivar (Gao et al., 1998; Sweetlove et al., 1999; Vrinten and Nakamura, 2000; Nakkanong et al., 2012). Total starch and amylase contents were measured in Zhewan 1 and Zhongwan 6 during seed development. As shown in Figure 7A, the total starch and amylose contents increased during seed development and reached a maximum at 30 DAP in both cultivars. Notably, grain pea accumulated a significantly higher level of starch compared to vegetable pea in almost all stages of seed development (Figure 7A). The total starch content at 30 DAP in the pea seeds of Zhewan 1 and Zhongwan 6 was approximately 11.42 and 28.03%, respectively (Figure 7A).

Together with the soluble sugar data obtained in these two cultivars, a negative correlation of seed starch and sugar content was observed in grain pea and vegetable pea. Sink organs such as developing seeds import sucrose, which is cleaved to provide carbon skeletons for the synthesis of other storage compounds commonly accumulated during late developmental stage (Santos-Mendoza et al., 2008). On the basis of our analysis and previous work (Heim et al., 1993), the dramatic increase in the amount of starch at late maturity is preceded by a parallel decrease in sucrose in pea. In plants, the regulation of starch metabolism is complex, but a simplified version of the starch synthesis pathway is shown in Figure 7B. Amylose is synthesized by AGPase (ADP-glucose pyrophosphorylase) and granule-bound starch synthase (GBSS), while amylopectin is synthesized by the coordinated actions of AGPase, soluble starch synthase (SS), starch branching enzyme (SBE), and starch debranching enzyme (DBE) (Tetlow, 2006). There are two classes of DBE in higher plants, which are the pullulanase-type DBE (PUL) and the isoamylase-type DBE (ISA) (Nakamura, 2002). To understand starch biosynthesis in vegetable pea and grain pea, all the unigenes involved in starch metabolism were selected, and the relative expression levels were analyzed by RNA-Seq and qRT-PCR. AGPase is a key regulatory allosteric enzyme that is involved in starch biosynthesis in higher plants (Jin et al., 2005). Previous studies using mutant lines with reduced activities of AGPase found a reduction in the amounts of both amylose and amylopectin and total starch in *Arabidopsis* and in pea (Smith et al., 1989; Ekkehard Neuhaus and Stitt, 1990). In both Zhewan 1 and Zhongwan 6 seeds, AGPase exhibited a bell-shaped expression pattern, which increased from the early stage to the middle stage of seed development and then decreased in the later stage. However, the highest transcript levels of AGPase were at 20 and 25 DAP in Zhongwan 6 and Zhewan 1 seeds, respectively (Figure 7C). The higher accumulation of starch in Zhongwan 6 than in Zhewan 1 seeds can be correlated with the transcript levels of AGPase that were vigorously expressed at the early phase of development in Zhongwan 6 seeds. In pea seeds, amylopectin is the main component of starch. As shown in Figure 7C, the expression patterns of SBE, DBE, PUL and ISA, which are involved in amylopectin synthesis, as well as GBSS, which is responsible for amylose synthesis, showed similar patterns in the two types of pea; however, the transcript levels were much higher in the Zhongwan 6 than in the Zhewan 1 seeds during development (Figure 7C). These results suggest that the higher expression levels of starch biosynthesis related genes in Zhongwan 6 seeds trigger more starch accumulation than in Zhewan 1 seeds.

Previous studies have reported that both mutations of SBE and AGPase cause a reduction in starch content and consequently an increase in sugars during embyro development in pea (Hedley et al., 1986; Hylton and Smith, 1992; Lloyd et al., 1996). Similarly, transgenic potato tubers with reduced AGPase activity stored large amounts of sugars instead of starch (Müller-Röber et al., 1992). These reports are in line with our results that a negative correlation of seed starch and sugar content exists in Zhewan 1 and Zhongwan 6. Sugar and starch metabolism are known to be related in plants (Borisjuk et al., 2004), and our data suggest that an alteration in the levels of an enzyme involved in the starch synthesis pathway leads to an alteration of the level of sugar, thus resulting in higher levels of sucrose in vegetable pea seed than grain pea seed.

### Response of transcription factors at early and late seed developmental stages of Zhewan 1 and Zhongwan 6

Transcription factors (TFs) are crucial regulatory proteins that mediate transcriptional regulation. Many previous studies have been conducted to identify the TFs responsible for controlling seed development (Le et al., 2010). Still, most of the TFs involved in seed development are not yet known, particularly in non-model species. Therefore, we analyzed TF gene expression at early and late seed developmental stages of the two pea cultivars. We identified 23 and 32 TFs in Zhewan 1 and Zhongwan 6 seeds, respectively, classified into 10 and 11 TF families, which were significantly differentially regulated during the early and late stages of seed development (Supplementary Figure S9; Supplementary Table S16). When combined, transcriptome expression profiling revealed that approximately 14 TF families, including bHLH, bZIP, ERF, GATA, LBD, NAC, GRAS, MYB, NF-X1, NF-YC, HB, B3, EIL, and HSF, were implicated in the regulation of seed development, which indicates that seed development is governed by complex transcriptional regulatory networks. Remarkably, the largest TF family regulating seed development was represented by ERF. ERF genes belong to the large AP2/ERF multigene family and have been shown to play a role in plant development, including in seed maturation (Vom Endt et al., 2002). Additionally, the homologs of some homeobox genes and NAC genes were down-regulated at the late developmental stage compared with the early stage, implying that they might play important roles for seed development in pea, particularly in the early stage. In *Arabidopsis*, homeobox genes are involved in the regulation of polarity establishment, embryo patterning and initiation, and maintenance of the SAM (shoot apical meristem); NAC TFs are also important at this stage because they work in coordination with the homeobox genes (Agarwal et al., 2011). In addition, a putative LBD showed dramatically increased expression at 25 DAP compared with 10 DAP in both cultivars. LBD proteins are plant-specific TFs that function in growth and development, and are also involved in regulating the ripening of fruit through interactions with EXPANSIN (Ba et al., 2014). However, the regulatory role of LBDs in seed development remains unknown.

We further analyzed the different regulatory TFs in these two cultivars. In total, 4 and 10 TFs were differentially regulated at 25 DAP and 10 DAP, respectively, between Zhewan 1 and Zhongwan 6 seeds (Supplementary Table S16). FUS3, which is a B3 domain-containing transcription factor and a key regulator of embryo development (Baumlein et al., 1994), was up-regulated by approximately 25-fold at 25 DAP compared with 10 DAP in the Zhewan 1 seeds. Moreover, FUS3 was up-regulated by approximately four-fold in the Zhewan 1 seeds compared with the Zhongwan 6 seeds at 25 DAP. Previous studies have reported that FUS3 functions primarily or exclusively as a transcriptional activator during embryogenesis (Luerssen et al., 1998). These results suggested that FUS3 could be a direct regulator of pea seed filling; however, the mechanism requires further study. When combined, our results indicated that TF gene expression undergoes significant changes during seed development to construct a complex regulatory network, and that the regulatory role of the TFs is somewhat different between vegetable and grain peas. The differentially expressed TF genes uncovered in our study should provide an important starting point for understanding how gene activity is coordinated during seed development in grain pea and vegetable pea. These TFs probably play an active role in storage product accumulation; however, the specific role of seed-regulated TF genes and their possible targets remains to be determined.

## Conclusions

Studying the molecular mechanisms underlying seed development is a difficult but important area of research in plant biology. Using NGS technologies, we provide here the first overview of transcriptome dynamics in different stages of seed development in vegetable pea and grain pea. Our results illustrate that transcriptional control during pea seed development is a highly coordinated process. We found much evidence demonstrating that the metabolism of carbohydrates, amino acids, and other contents for storage are transcriptionally regulated from the early to late developmental stages in pea seeds. Furthermore, we provide a detailed comparison of gene expression between vegetable pea and grain pea, which differ in soluble sugar and starch. Our results reveal that the differential expression of most genes participating in sugar and starch biosynthesis at late development stages results in a negative correlation between soluble sugar and starch biosynthetic flux in vegetable pea and grain pea seeds. The information obtained from this study serve as a powerful foundation for understanding candidate genes that may have crucial roles in regulating and coordinating the complicated metabolisms and regulatory networks during seed development, and also provides a resource for the improvement of vegetable pea and grain pea breeding.

## Author contributions

Conceived and designed the experiments: NL, GZ, YG. Performed the experiments: NL, GZ, SX, QH. Analyzed the data: NL, WM. Contributed reagents/materials/analysis tools: NL, GZ, SX. Wrote the paper: NL, YG.

### Conflict of interest statement

The authors declare that the research was conducted in the absence of any commercial or financial relationships that could be construed as a potential conflict of interest.

## Supplementary material

Supplementary Table S1 **Primers for qRT-PCR analysis**.

Supplementary Table S2 **Unigenes were annotated with *Medicago truncatula* genome**.

Supplementary Table S3 **Unigenes were aligned with recently announced public pea (cv. “Cameor”) Unigene set (http://bios.dijon.inra.fr/FATAL/cgi/pscam.cgi)**.

Supplementary Table S4 **GO annotation of pea unigenes**.

Supplementary Table S5 **KEGG pathway annotation of pea unigenes**.

Supplementary Table S6 **Differentially expressed genes between Zhewan 1–10 and Zhewan 1–25**.

Supplementary Table S7 **Differentially expressed genes between Zhongwan 6–10 and Zhongwan 6–25**.

Supplementary Table S8 **Differentially expressed genes between Zhewan 1–10 and Zhongwan 6–10**.

Supplementary Table S9 **Differentially expressed genes between Zhewan 1–25 and Zhongwan 6–25**.

Supplementary Table S10 **Differentially expressed genes that overlapping in the comparisons of Zhewan 1–10 vs Zhewan 1–25 and Zhongwan 6–10 vs Zhongwan 6–25**.

Supplementary Table S11 **List of unigenes assigned for MapMan analysis that significant differentially expressed between Zhewan 1 and Zhongwna 6 seeds at 10 and 25 DAP**.

Supplementary Table S12 **Genes with different expression involved in amino acid metabolism**.

Supplementary Table S13 **Genes with different expression involved in photosynthesis**.

Supplementary Table S14 **Genes with different expression involved in phytohormone biosynthesis and signaling transduction**.

Supplementary Table S15 **Genes with different expression involved in sugar metabolism at 10 and 25 DAP of Zhewan 1 and Zhongwan 6**.

Supplementary Table S16 **Differentially expressed transcription factors in Zhewan 1 and Zhongwan 6 at 10 DAP and 25 DAP**.

Supplementary Figure S1 **COG annotation of putative proteins**.

Supplementary Figure S2 **The top 500 highly expressed genes differing between 10 and 25 DAP in Zhewan 1 and Zhongwan 6 developing seeds**.

Supplementary Figure S3 **Coefficient analysis between gene expression ratios obtained from the RNA-seq and the qRT-PCR data**. Scatter plot shows expression change (log_2_-fold) measured by RNA-seq and by qRT-PCR analysis of the selected genes. A linear trend line is shown. ^***^indicates a significant difference at *p* ≤ 0.001.

Supplementary Figure S4 **Functional categorization of genes differentially expressed at 10 and 25 DAP in Zhewan 1 and Zhongwan 6 seeds based on biological process of GO**.

Supplementary Figure S5 **Functional categorization of genes differentially expressed at 10 and 25 DAP in Zhewan 1 and Zhongwan 6 seeds based on KEGG**.

Supplementary Figure S6 **MapMan pathways depicting regulation of vegetable pea and grain pea seed development**. Genes differentially regulated in the “metabolism overview” and “regulatory pathways overview” for 25 DAP seeds as compared to10 DAP seeds in Zhewan 1 **(A,C)** and Zhongwan 6 **(B,D)**, respectively. The scale depicts level of expression, with blue being high and red being low. TCA is symbolized by tricarboxylic acid, CHO by carbohydrate, OPP by oxidative pentose phosphate, auxin by IAA, abscissic acid by ABA, brassinosteroids by BA, salicylic acid by SA, and gibberellic acid by GA.

Supplementary Figure S7 **MapMan display of genes differentially regulated in the “metabolism overview” and “regulatory pathways overview” between Zhewan 1 and Zhongwan 6 seeds at 10 DAP (A,C) and 25 DAP (B,D), respectively**. Scale depicts level of expression; blue being high; and red being low.

Supplementary Figure S8 **Differential expression of genes for amino acid biosynthesis (A), photosynthesis (B), and hormones biosynthesis and signal transduction (C) in Zhewan 1 and Zhongwan 6 seeds at 10 and 25 DAP**.

Supplementary Figure S9 **Distribution of TFs differentially expressed between Zhewan 1 and Zhongwan 6 seeds at 10 and 25 DAP**.
