# Supplementary figures and images for: Comparative Transcriptomic Analyses of Vegetable and Grain Pea (Pisum sativum L.) Seed Development
Source: Front Plant Sci. 2015 Nov 25;6:1039. doi: 10.3389/fpls.2015.01039 (PMC4658420; doi:10.3389/fpls.2015.01039)

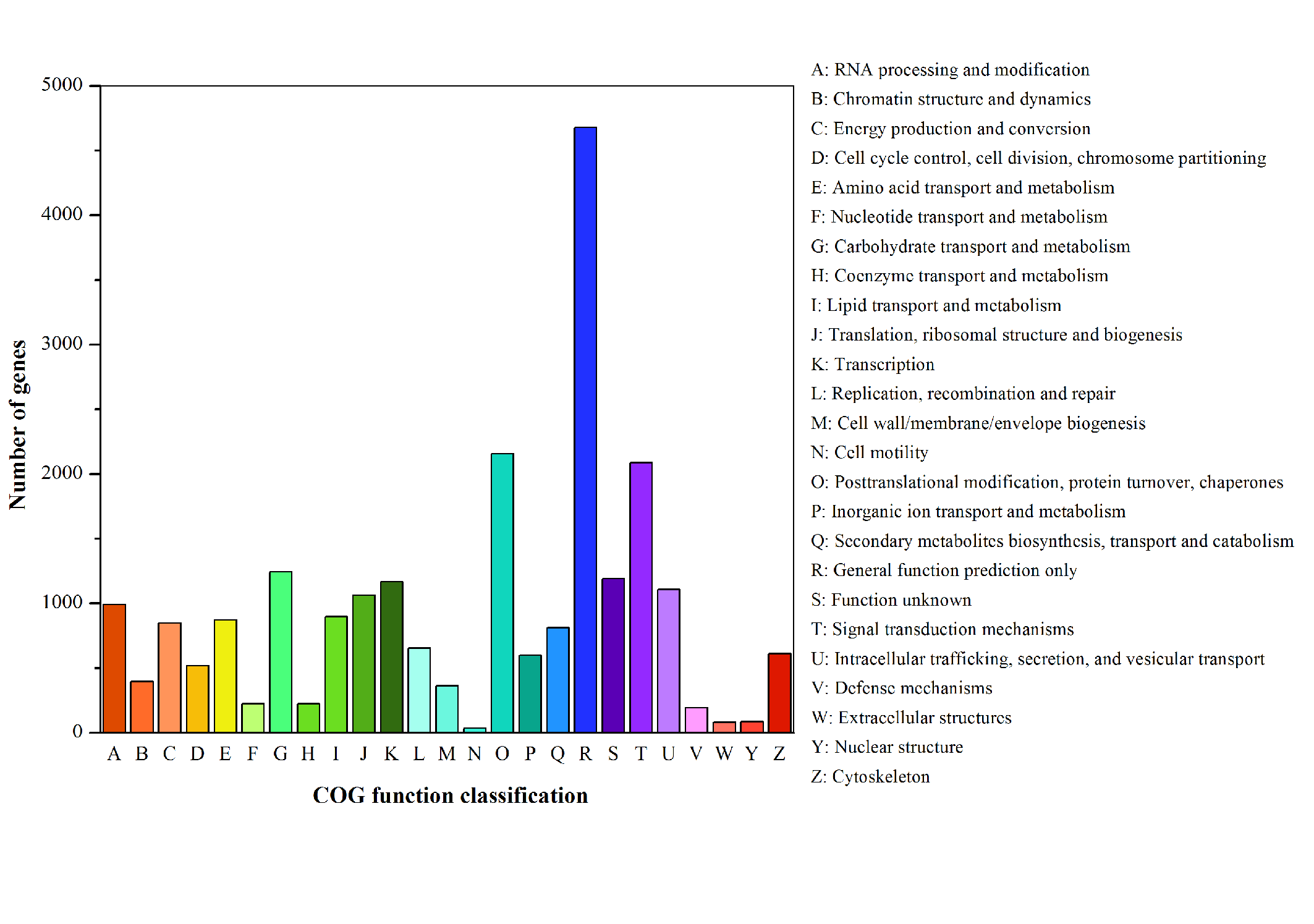

Supplement: Supplementary Figure S1 — COG annotation of putative proteins. [file Image1.TIF]

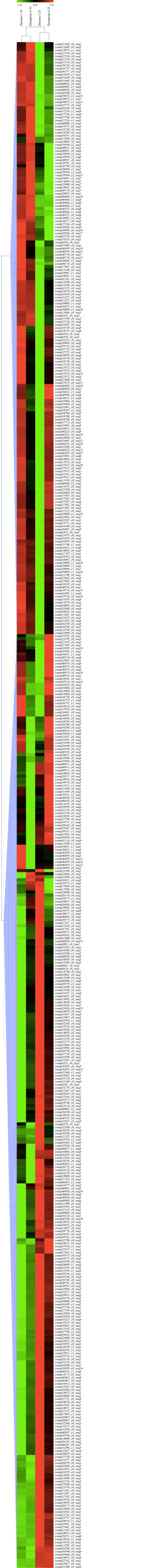

Supplement: Supplementary Figure S2 — The top 500 highly expressed genes differing between 10 and 25 DAP in Zhewan 1 and Zhongwan 6 developing seeds. [file Image2.PDF]

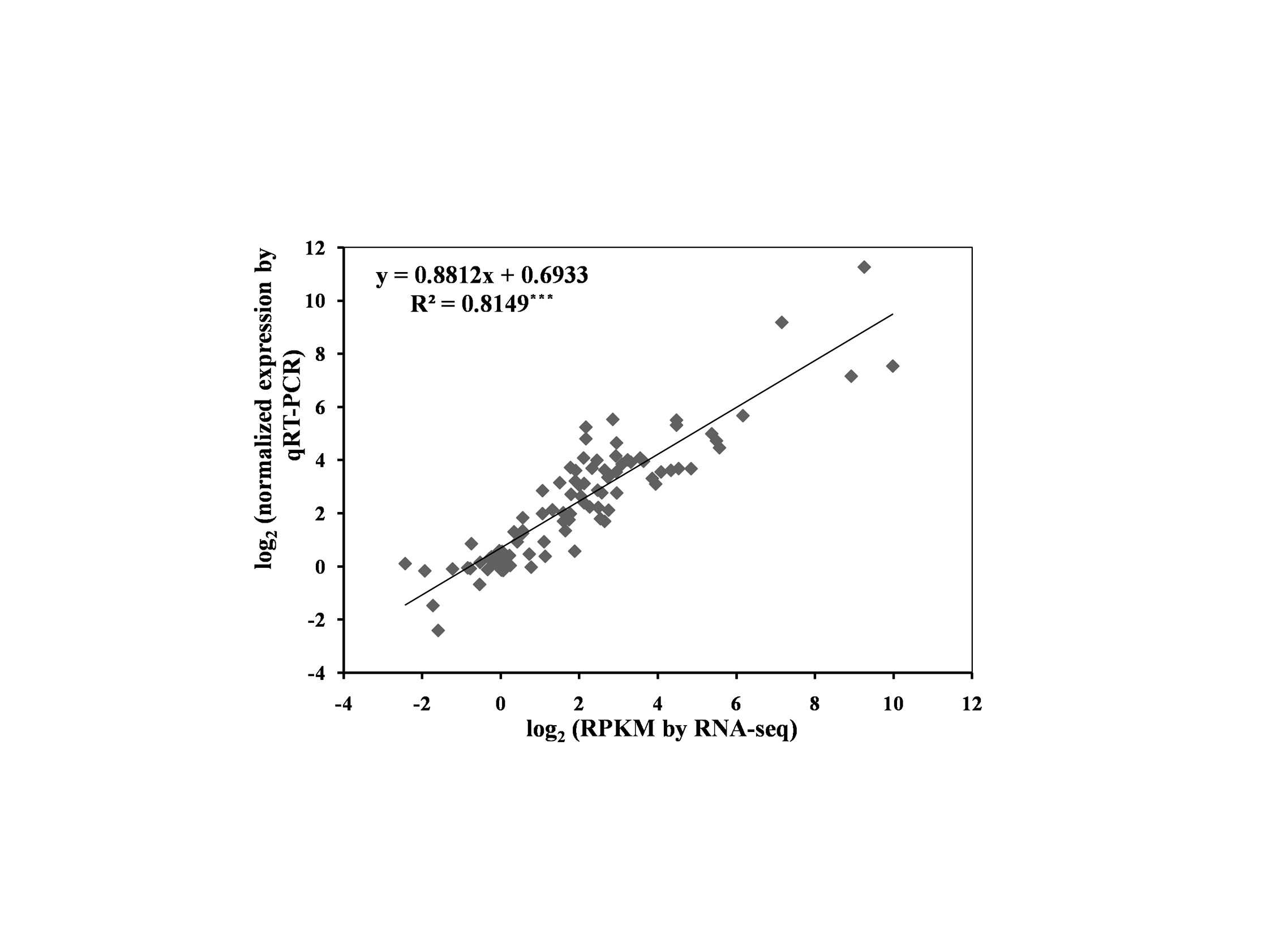

Supplement: Supplementary Figure S3 — Coefficient analysis between gene expression ratios obtained from the RNA-seq and the qRT-PCR data. Scatter plot shows expression change (log2-fold) measured by RNA-seq and by qRT-PCR analysis of the selected genes. A linear trend line is shown. ***indicates a significant difference at p ≤ 0.001. [file Image3.TIF]

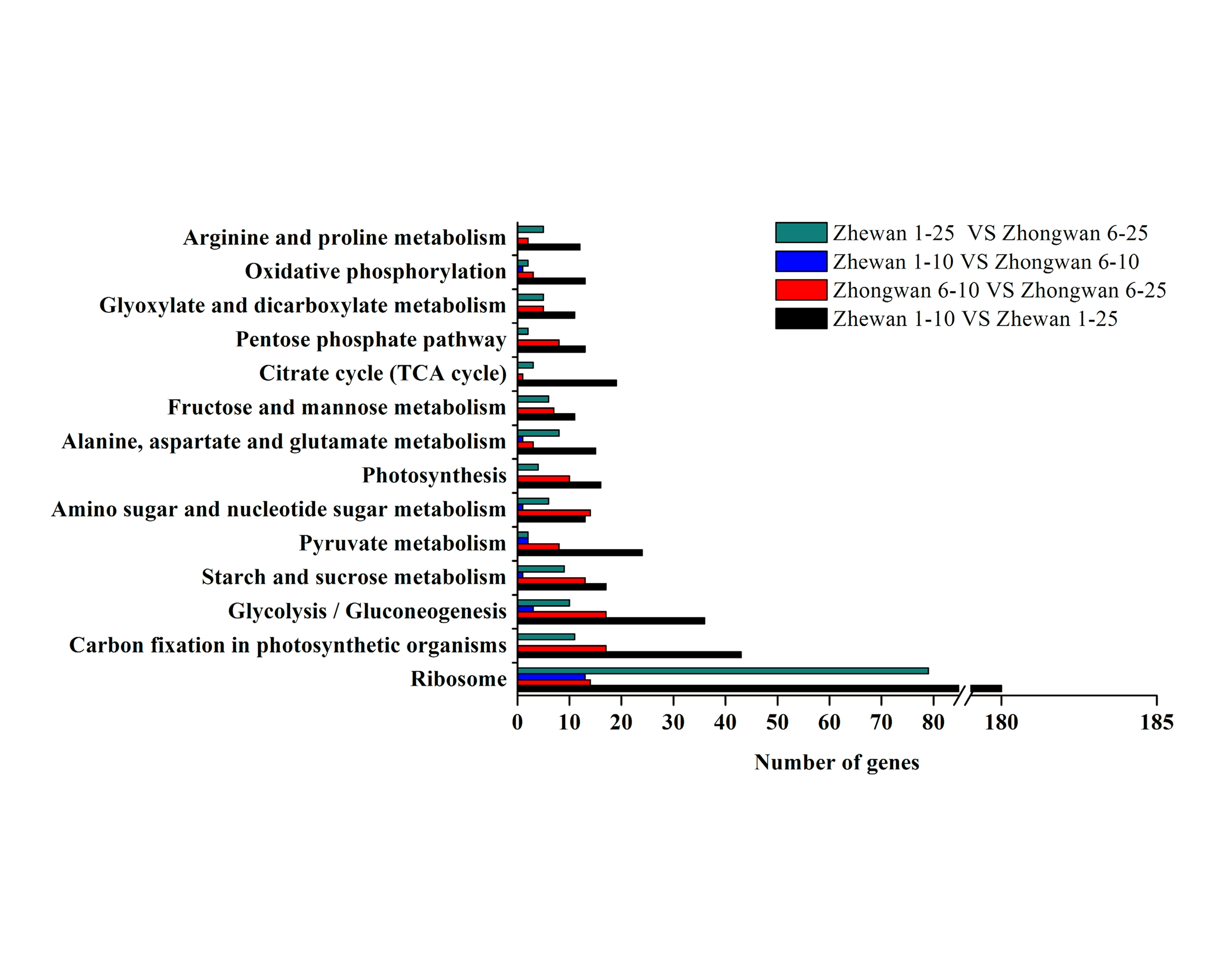

Supplement: Supplementary Figure S4 — Functional categorization of genes differentially expressed at 10 and 25 DAP in Zhewan 1 and Zhongwan 6 seeds based on biological process of GO. [file Image4.TIFF]

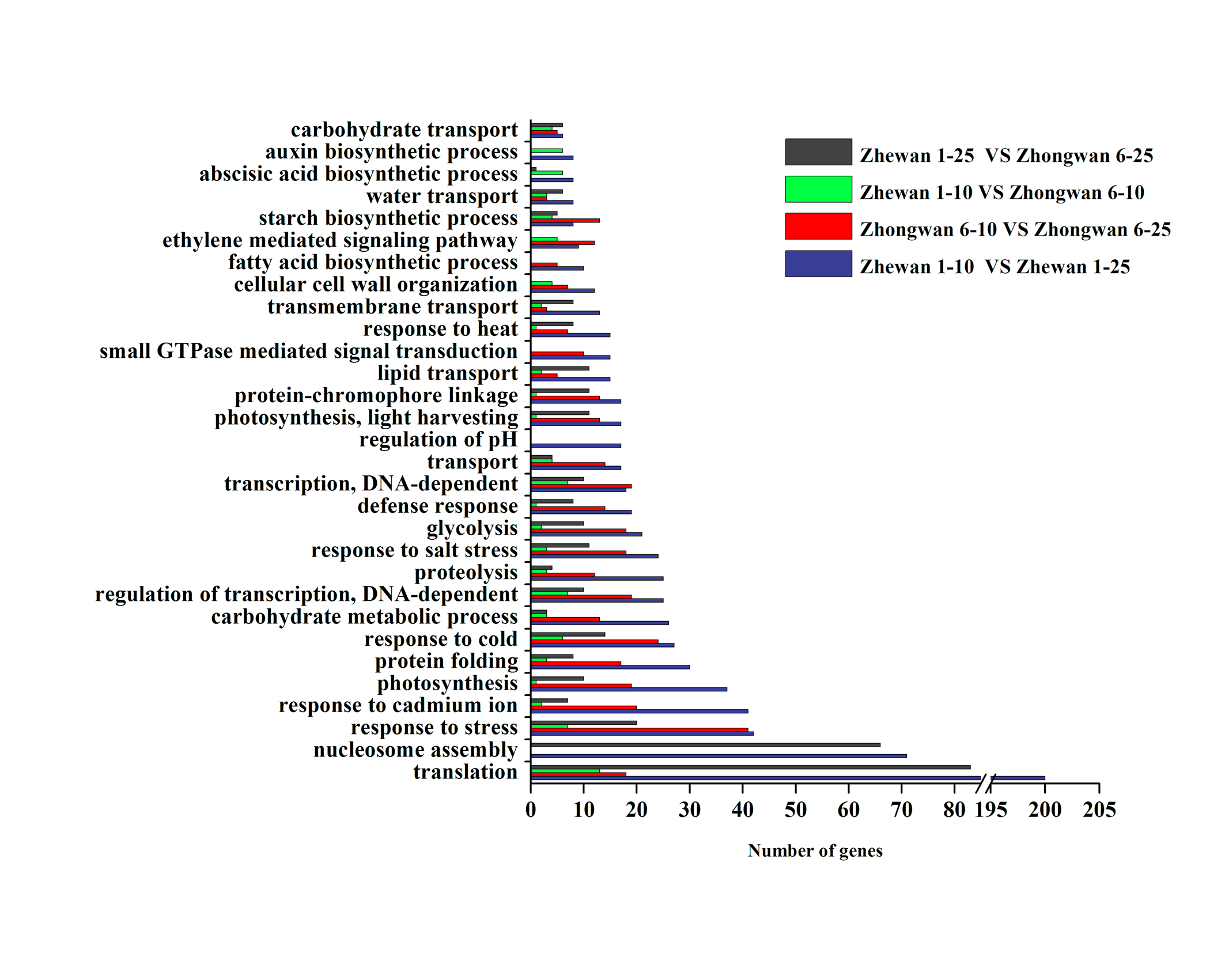

Supplement: Supplementary Figure S5 — Functional categorization of genes differentially expressed at 10 and 25 DAP in Zhewan 1 and Zhongwan 6 seeds based on KEGG. [file Image5.TIFF]

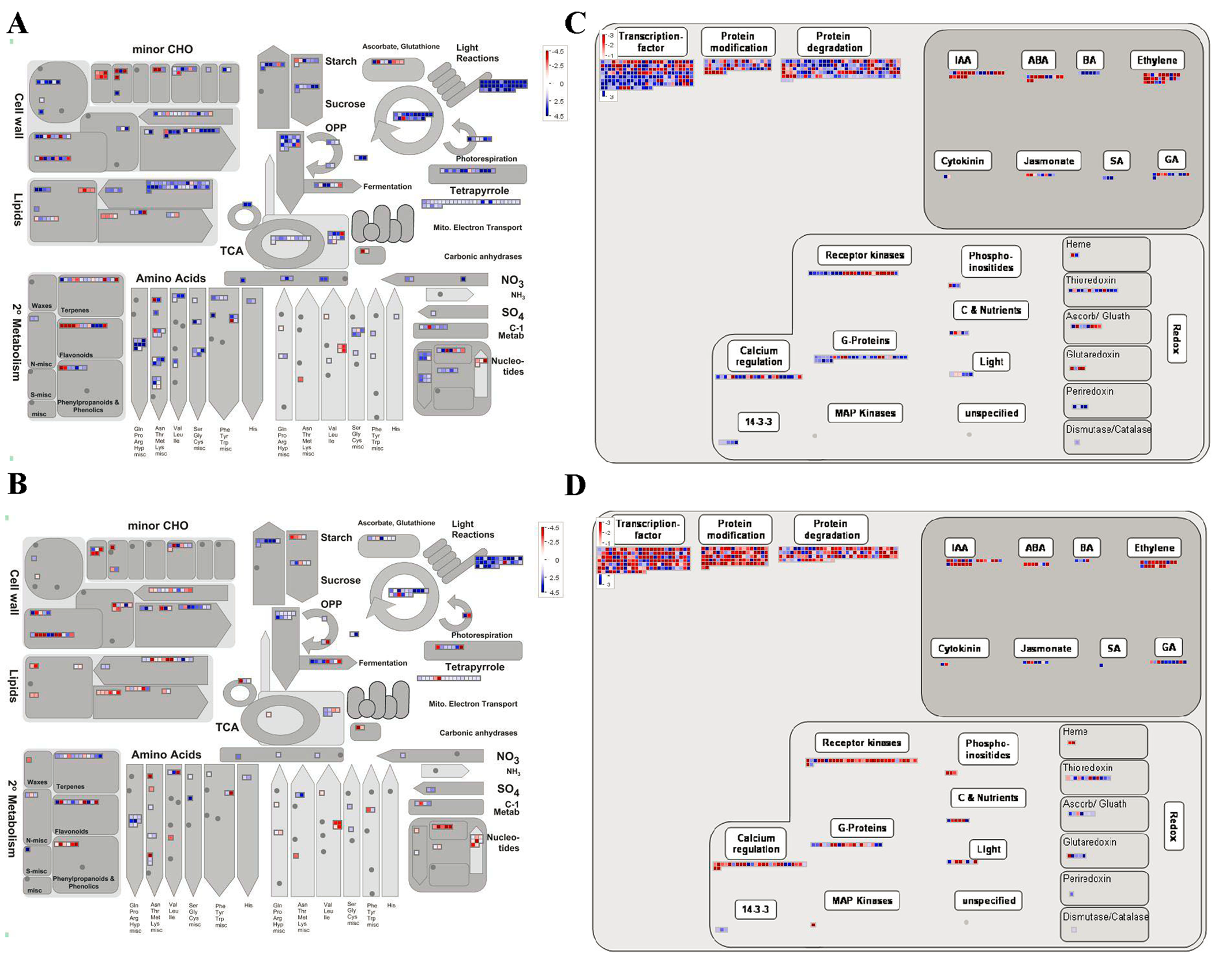

Supplement: Supplementary Figure S6 — MapMan pathways depicting regulation of vegetable pea and grain pea seed development. Genes differentially regulated in the “metabolism overview” and “regulatory pathways overview” for 25 DAP seeds as compared to10 DAP seeds in Zhewan 1 (A,C) and Zhongwan 6 (B,D), respectively. The scale depicts level of expression, with blue being high and red being low. TCA is symbolized by tricarboxylic acid, CHO by carbohydrate, OPP by oxidative pentose phosphate, auxin by IAA, abscissic acid by ABA, brassinosteroids by BA, salicylic acid by SA, and gibberellic acid by GA. [file Image6.TIF]

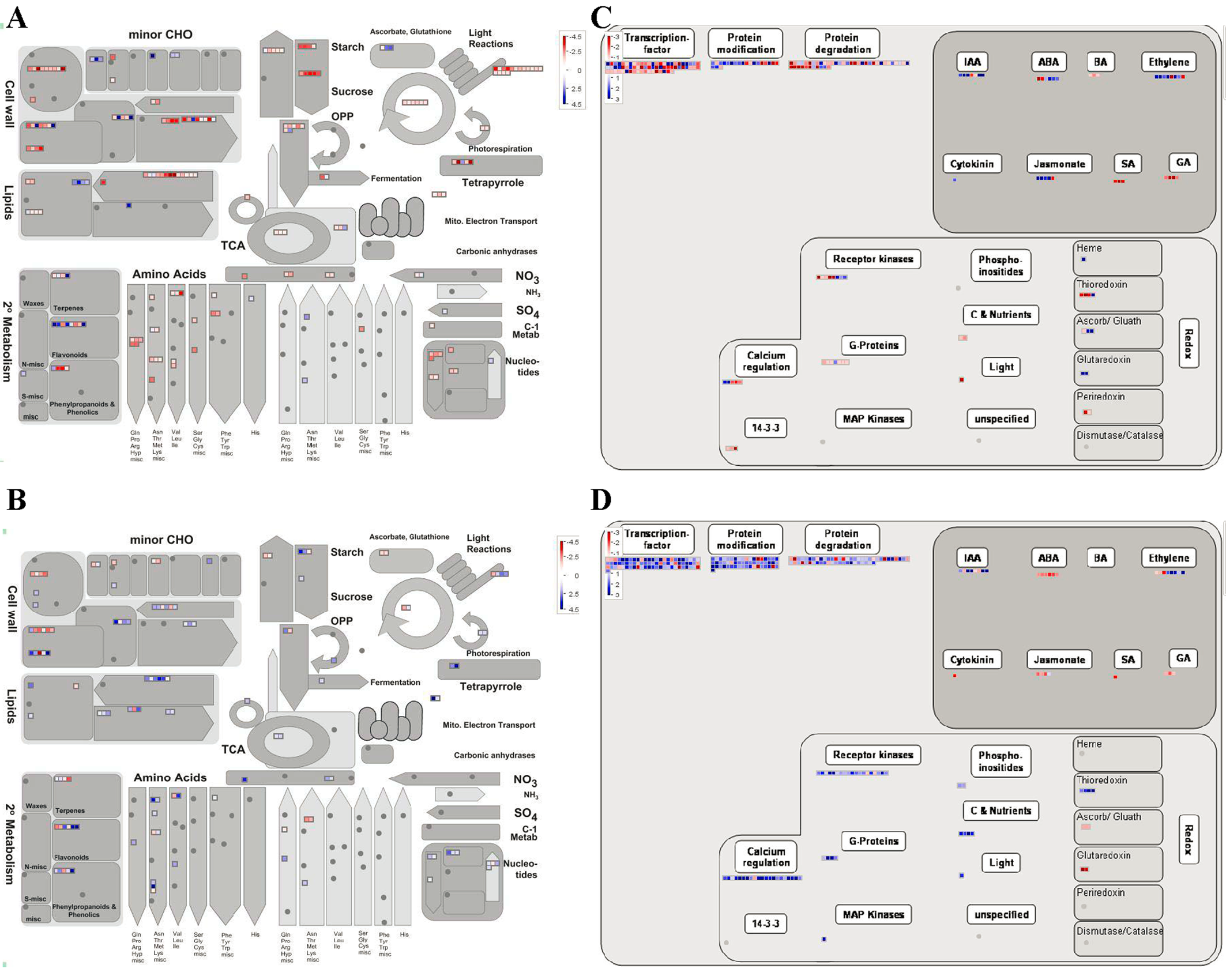

Supplement: Supplementary Figure S7 — MapMan display of genes differentially regulated in the “metabolism overview” and “regulatory pathways overview” between Zhewan 1 and Zhongwan 6 seeds at 10 DAP (A,C) and 25 DAP (B,D), respectively. Scale depicts level of expression; blue being high; and red being low. [file Image7.TIF]

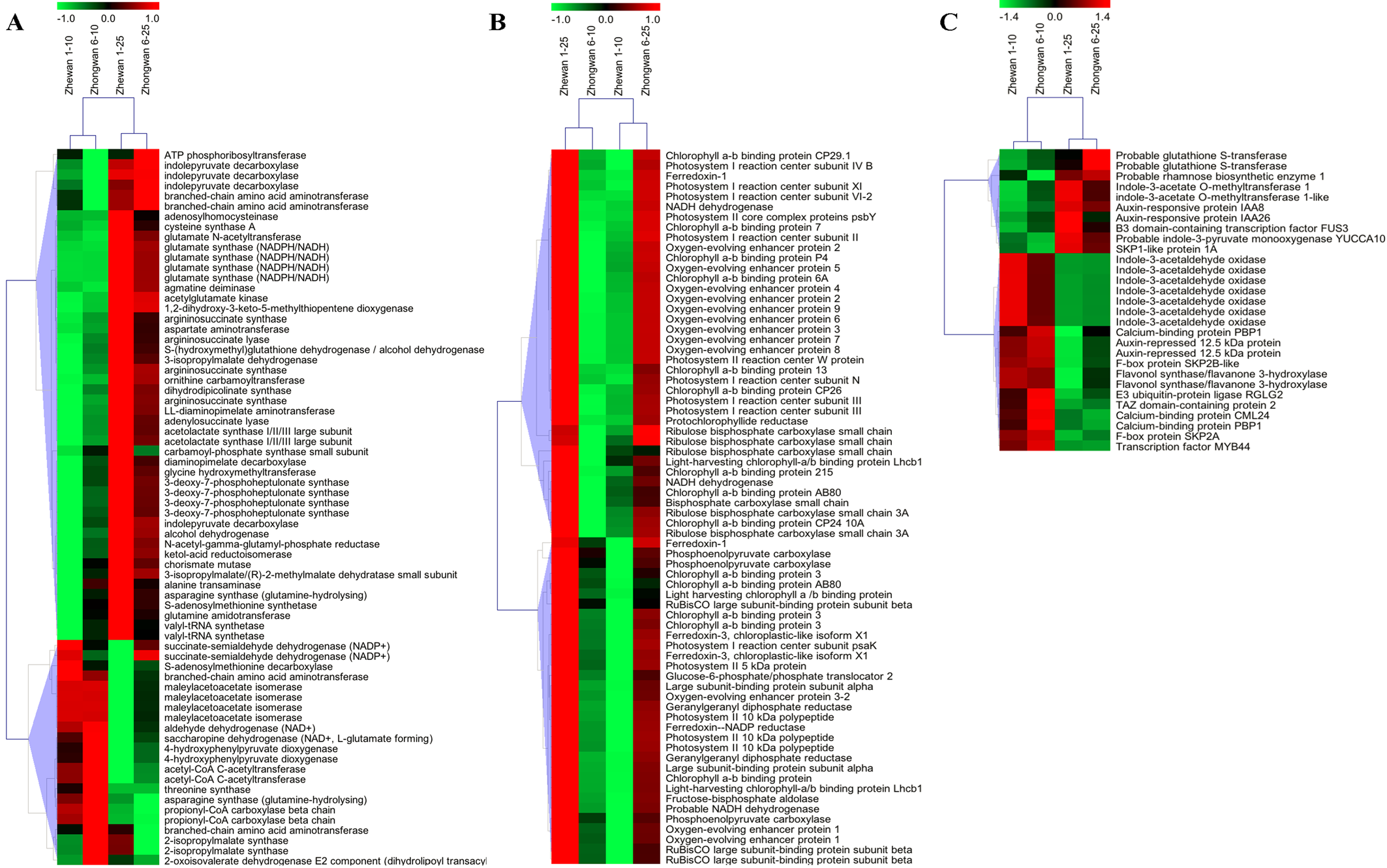

Supplement: Supplementary Figure S8 — Differential expression of genes for amino acid biosynthesis (A), photosynthesis (B), and hormones biosynthesis and signal transduction (C) in Zhewan 1 and Zhongwan 6 seeds at 10 and 25 DAP. [file Image8.TIF]

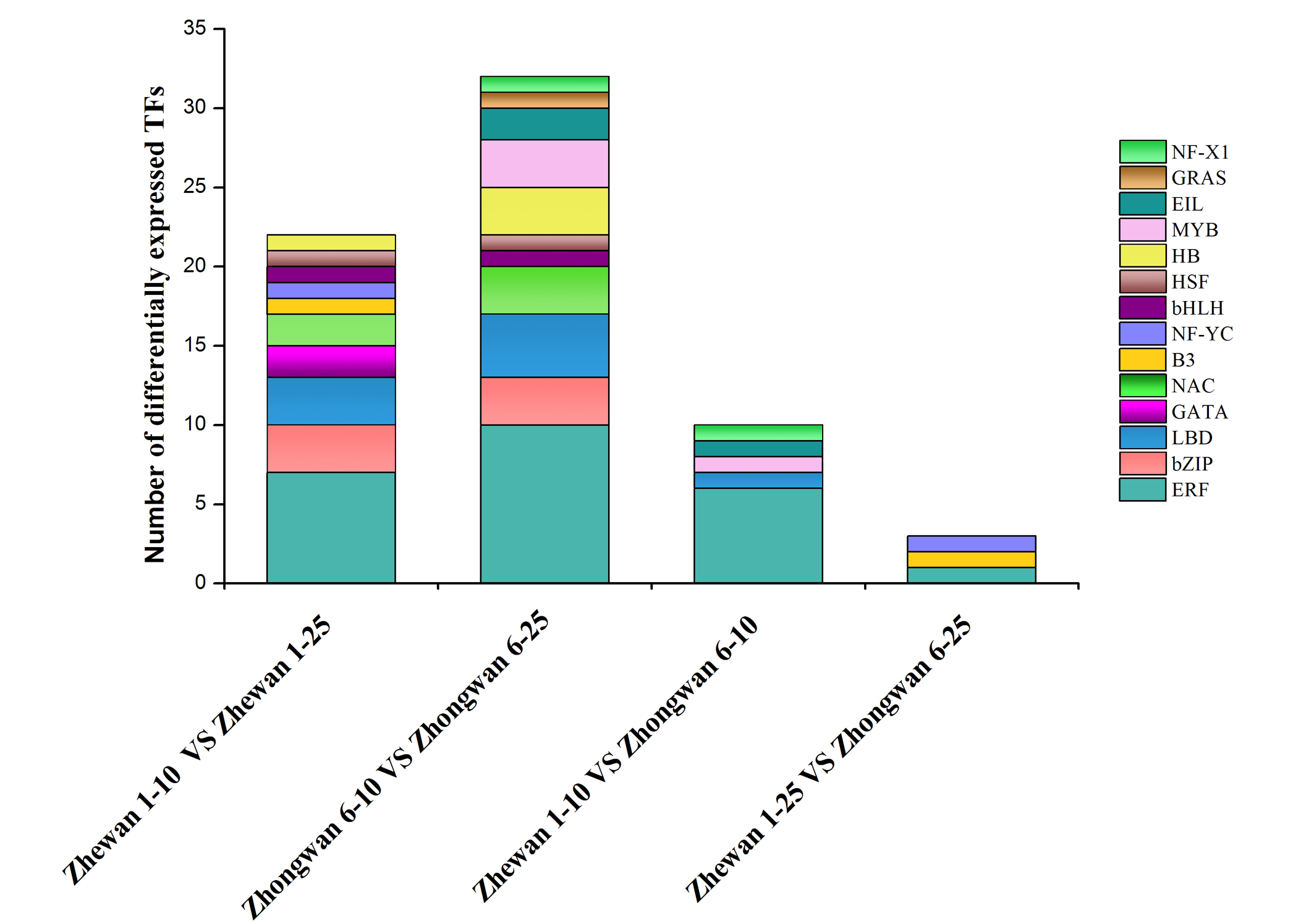

Supplement: Supplementary Figure S9 — Distribution of TFs differentially expressed between Zhewan 1 and Zhongwan 6 seeds at 10 and 25 DAP. [file Image9.TIF]
